# A unique case of human Zika virus infection in association with severe liver injury and coagulation disorders

**DOI:** 10.1038/s41598-017-11568-4

**Published:** 2017-09-12

**Authors:** Yanhua Wu, Xiaoyun Cui, Na Wu, Rui Song, Wei Yang, Wei Zhang, Dongying Fan, Zhihai Chen, Jing An

**Affiliations:** 1 Department of Microbiology and Parasitology, School of Basic Medical Sciences, Capital Medical University, Beijing, China; 2 Department of Experimental Animal, Capital Medical University, Beijing, China; 3 Department of Infectious Disease, Beijing Ditan Hospital, Capital Medical University, Beijing, China; 4 Center of Epilepsy, Beijing Institute for Brain Disorders, Beijing, China

## Abstract

Zika virus (ZIKV) has caused major concern globally due to its rapid dissemination and close association with microcephaly in children and Gullian-Barr syndrome in adults. In this study, we identified a patient returned from Cambodia who experienced high fever, chill and myalgia. Lab tests discovered sign of severe liver injury including significantly elevated serum transaminases’ level, decreased serum albumin level, and markedly increased levels of lactic dehydrogenase, alpha-hydroxybutyric dehydrogenase and creatine kinase in serum. Moreover, severe thrombocytopenia and altered blood levels of fibrinogen and fibrinogen degradation product were also observed, indicating the existence of clotting disorders. A ZIKV strain clustered into the Asian lineage was isolated from the patient’s serum. When inoculated into suckling mice, this virus significantly retarded mouse body-weight gain and caused 70% mortality. Our results demonstrate a close association between ZIKV and severe liver injury and coagulation disorders and suggest that clinicians should be aware of compatible symptoms in patients and manage them accordingly.

## Introduction

Zika virus (ZIKV) is a small, enveloped, single positive stranded RNA virus that belongs to the *Flavivirus* genus of the *Flaviviridae* family. ZIKV was first identified in a sentinel monkey in Uganda in 1947^1^ and first isolated in human in 1952^2^. It was neglected previously because most ZIKV infected individuals were asymptomatic, only 20% of patients developed a self-limited acute febrile illness with mild symptoms and signs including fever, myalgia, sore throat, conjunctivitis and maculopapule rash, that resolved within 1–2 weeks^3–6^. These clinical manifestations are very similar to that of dengue fever (DF), caused by dengue virus (DENV), another important member of *Flaviviruses*. However, in recent years, more serious complications such as microcephaly malformations in newborns and Guillain–Barré syndrome in adults have been reported in ZIKV-infected individual. Furthermore, some unusual clinical signs such as transient hearing loss, vomiting, retinal lesions, severe abdominal pain and cardiovascular complications were also observed in patients with ZIKV infection^7–10^.

Here we described a ZIKV infected 29-year-old Chinese man who was previously healthy until having had a business trip to Cambodia for 7 days. Upon returning, he claimed to have been bitten by mosquitoes in Cambodia, and experienced high fever (40.4 °C), chills, fatigue, sore throat and myalgia. Serial blood samples were obtained from him for routine clinical laboratory tests, and identification of specific pathogens. These tests confirmed that the patient had ZIKV infection. Concomitantly, he was showed clinical signs of severe liver injury and coagulation disorders; the former had never been reported before, and the latter had only 16 reported cases worldwide. Our results suggest that clinicians should be vigilant of these usual complications of ZIKV infection, and manage them promptly.

## Results

### Changes in routine clinical parameters in the patient

A battery of routine blood examinations was performed. Results showed that his total counts of white blood cell (WBC), neutrophils (NE) and lymphocytes (LY) were transiently decreased on D6 and D7. WBC and NE counts returned to normal levels from D8. After a slight increase on D8 and D9, LY returned to normal range on D10. During hospitalization, his other routine clinical parameters such as basophilic granulocyte (BASO) and monocyte (MO), red blood cell (RBC) count and hemoglobin level (HGB) maintained at normal range (Table 1).

**Table 1:** Changes of parameters of blood routine tests in the patient.

	D6*	D7*	D8*	D9*	D10*	D11*	D12*	Reference value
WBC (×10^9^/L)	2.28	3.00	6.83	6.98	5.13	4.72	3.55	3.5~9.5
NE (×10^9^/L)	1.34	1.31	2.96	2.55	1.96	1.86	1.64	1.8~6.3
LY (×10^9^/L)	0.83	1.08	3.26	3.52	2.54	2.42	1.35	1.1~3.2
MO (×10^9^/L)	0.09	0.51	0.41	0.62	0.50	0.39	0.28	0.1~0.6
EO (×10^9^/L)	0	0.03	0	0.04	0.01	0.03	0.01	0.02~0.52
BASO (×10^9^/L)	0.02	0.08	0.16	0.19	0.09	0.02	0.04	0~0.06
RBC (×10^12^/L)	5.02	4.59	5.23	4.49	4.7	4.56	4.44	4.00–5.50
HGB (g/L)	148	140	163.4	139.4	146.4	134.2	137	120–160

WBC = white blood cell; RBC = red blood cell; HGB = hemoglobin; LY = lymphocytes; MO = Monocyte; NE = Neutrophils; EO = eosinophils; BASO = basophilic granulocyte. *Days post fever onset.

**Figure 1:**
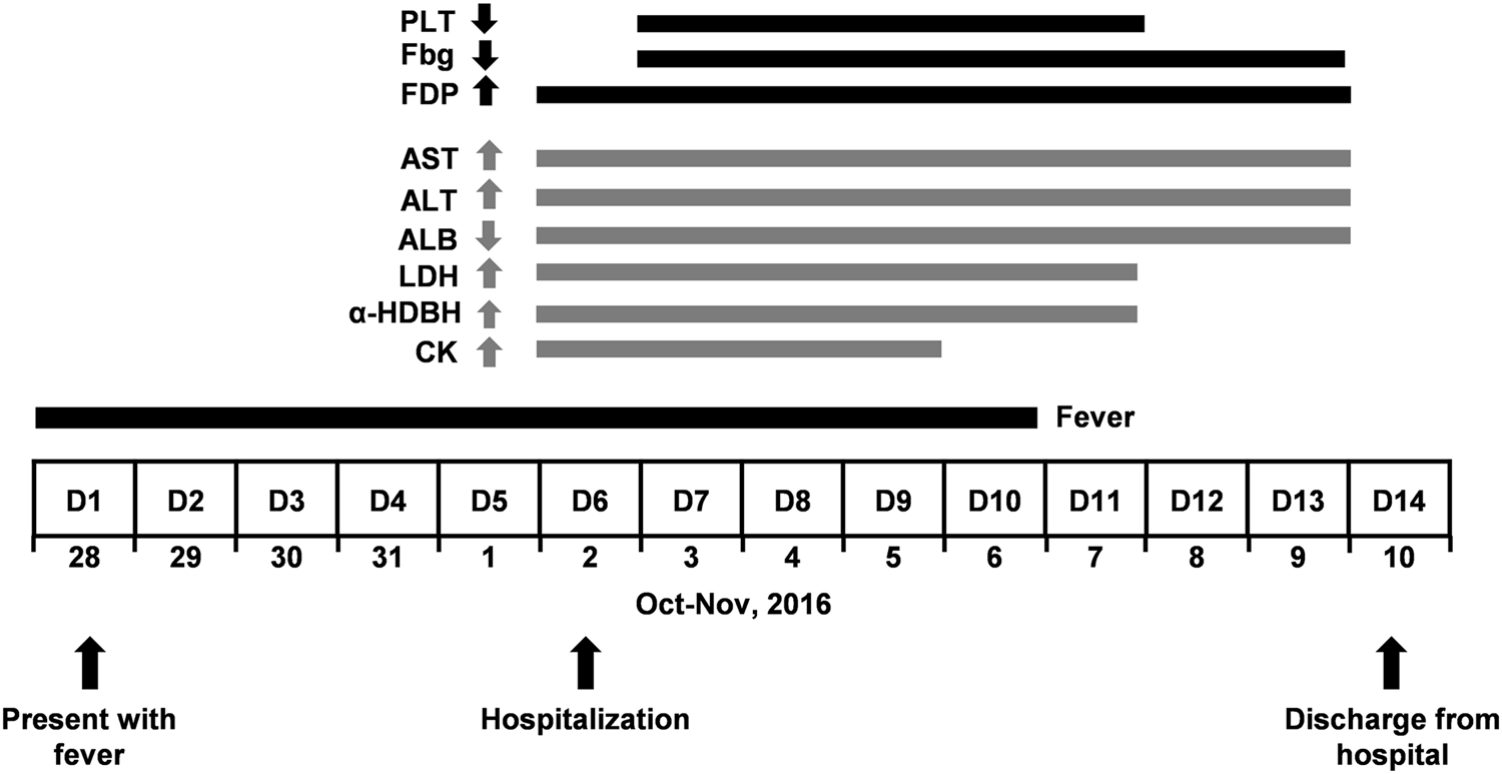
Timeline of clinical events. Body temperature was recorded daily. Changes of parameters reflecting coagulation and liver function were determined by routine blood examination and biochemistry tests from D6 to being discharged from the hospital.

Importantly, several parameters reflecting tissue or organ damage were also recorded (Fig. 1, Table 2). During the entire period of hospitalization, the patient had significantly elevated levels of aspartate aminotransferase (AST) (122.4–511.7 U/L), alanine aminotransferase (ALT) (225.8–329.6 U/L), lactic dehydrogenase (LDH) (909–1433 U/L) and alpha-hydroxybutyric dehydrogenase (α-HDBH) (792–931 U/L), but reduced albumin (ALB) concentrations (31.9–39.3 g/L). On D6 to D9, increased creatine kinase (CK) level (792–937 U/L) was also observed. The highest concentrations of AST (511.7 U/L) and ALT (329.6 U/L) were 12.8 and 6.6 folds higher than the upper limits of their respective normal range. The highest levels of LDH, α-HDBH and CK were 3–6 times higher than the upper limits of their normal range. These results indicate serious tissue injury, especially liver damage.

**Table 2:** Changes of several parameters reflecting coagulation and liver functions in the patient.

	D6*	D7*	D8*	D9*	D10*	D11*	D13*	Reference value
PLT (×10^9^/L)	128	22	20	24.4	37.4	63.4	194	125~350
Fbg (g/L)	1.38	1.47	1.15	1.21	1.57	1.62	1.83	1.70–4.00
FDP (mg/L)	33.4	38.7	26.19	16.47	17.21	14.48	5.66	0–5
AST (U/L)	356	511.7	331.5	265.7	372.6	241.6	122.4	15–40
ALT (U/L)	192	283	246.7	207.1	329.6	302.9	225.8	9–50
ALB (U/L)	36.2	39.3	31.9	31.9	32	32.3	32.5	40–55
LDH (U/L)	1601	1433	1161	909	1300	994	463	120–250
α-HDBH (U/L)	782	862	931	792	937	851	532	74–182
CK (U/L)	803	1103	645.3	359.6	192.8	83.5	NT	50–310

PLT = platelet; Fbg = fibrinogen; FDP = fibrin degradation product; AST = aspartate aminotransferase ALT = alanine aminotransferase; ALB = albumin; LDH = lactic dehydrogenase; α-HDBH = alpha-hydroxybutyric dehydrogenase; CK = creatine kinase. *Days post symptom onset. NT: Untested.

Concomitantly, blood coagulation parameters showed marked alterations: the platelet count decreased suddenly to 22 × 10^9^ platelets/L on D7 and remained at low levels between 20 × 10^9^/L and 63.4 × 10^9^ platelets/L during D8 to D11. It returned to normal range on D13 (125–350 × 10^9^ platelets/L) (Fig. 1, Table 2). Additionally, decreased fibrinogen (Fbg) levels and increased fibrin degradation product (FDP) levels in blood were also observed from D6 to D11 (Fig. 1, Table 2). The lowest level of Fbg was 1.15 g/L and the highest value of FDP was 38.7 mg/L. These changes are indicative of abnormal blood coagulation and anticoagulation pathways in the patient, although he had no obvious clinical sign of bleeding.

### Virus isolation and identification

**Figure 2:**
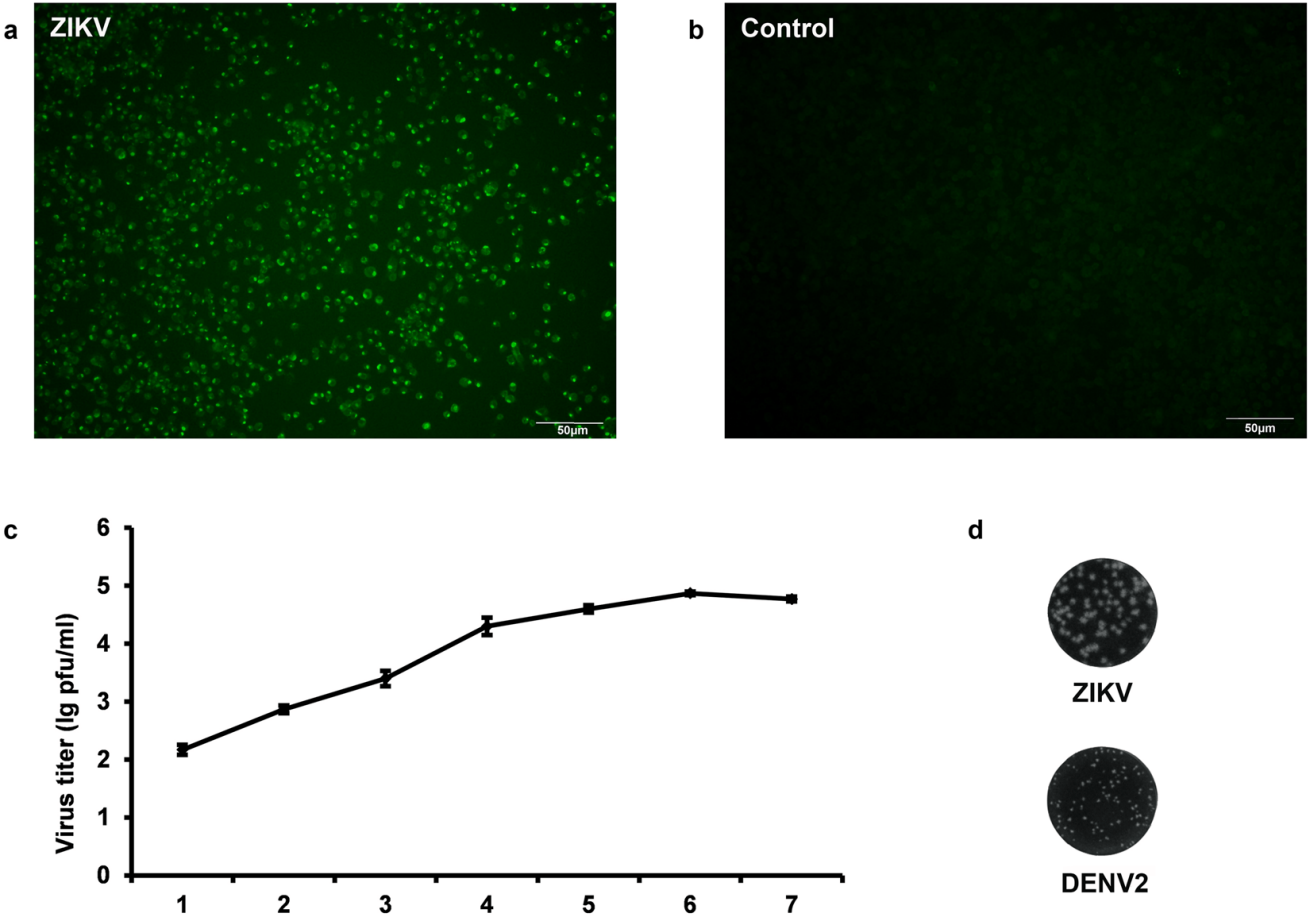
Virus identification and viral growth curve. (**a**) Virus infection was detected by immunofluorescence (IFA) using 1:200 diluted anti-ZIKV polyclonal antibody as primary antibody, followed by 1:500 diluted FITC-conjugated anti-mouse IgG as secondary antibody. (**b**) C6/36 cells without infection served as the control. (**c**) Virus growth curve in C6/36 cells at 1–7 dpi were determined by plaque assay on Vero cells under a 1.2% methylcellulose overlay medium. (**d**) Plaque of ZIKV on Vero cells at 5 dpi compared with that of DENV2 at 7 dpi.

ZIKV was isolated by sequential passage in C6/36 cells and named ZIKV-CCMU01 (GenBank NO.: MF036115). Although no apparent cytopathic effect developed in the infected C6/36 cells, retarded cell growth was observed on passages 3. RNA was extracted from infected C6/36 cells and quantitative real-time polymerase chain reaction (qRT-PCR) was positive for ZIKV, but negative for DENV and Chikungunya virus (CHIKV). Immunofluorescence (IFA) showed strong ZIKV antigen specific fluorescence in the cytoplasm (Fig. 2a). Viral replication kinetics in C6/36 cells was shown in Fig. 2c. At multiplicity of infection (MOI) of 1, the virus titer gradually increased to 2 × 10^4^ pfu/ml at 4 post infection (dpi) and reached peak level with a titer of 2 × 10^5^ pfu/ml at 6 dpi, and then decreased slightly to 6 × 10^4^ pfu/ml at 7 dpi (Fig. 2c). The virus could form plaques on Vero cells, and the plaque sizes at 5 dpi were larger than that of DENV2 at 7dpi (Fig. 2d). Serologic tests were positive for anti-ZIKV IgM and IgG on D6, but negative for anti-DENV and anti-CHIKV. Serum samples obtained on D6 and D12 had 50% plaque reduction (PRNT50) titers of 1:40 and 1:160 to ZIKV, respectively. These results confirmed that the patient was infected by ZIKV.

Furthermore, serologic tests showed that IgM antibodies to human cytomegalovirus (HCMV), herpes simplex virus type 1 (HSV-1), HSV-2, human immunodeficiency virus (HIV), hepatitis C virus (HCV), Epstein-Barr virus (EBV), and Treponema pallidum were all negative. IgG antibody to HBsAg (surface antigen of hepatitis B virus (HBV)) was positive as a result of HBV vaccination. The blood culture was also negative for bacteria and fungus. These results were consistent with an interpretation that most manifested symptoms and signs in this individual were a direct consequence of ZIKV infection.

### Genome sequencing and phylogenetic analysis of ZIKV-CCMU01

**Figure 3:**
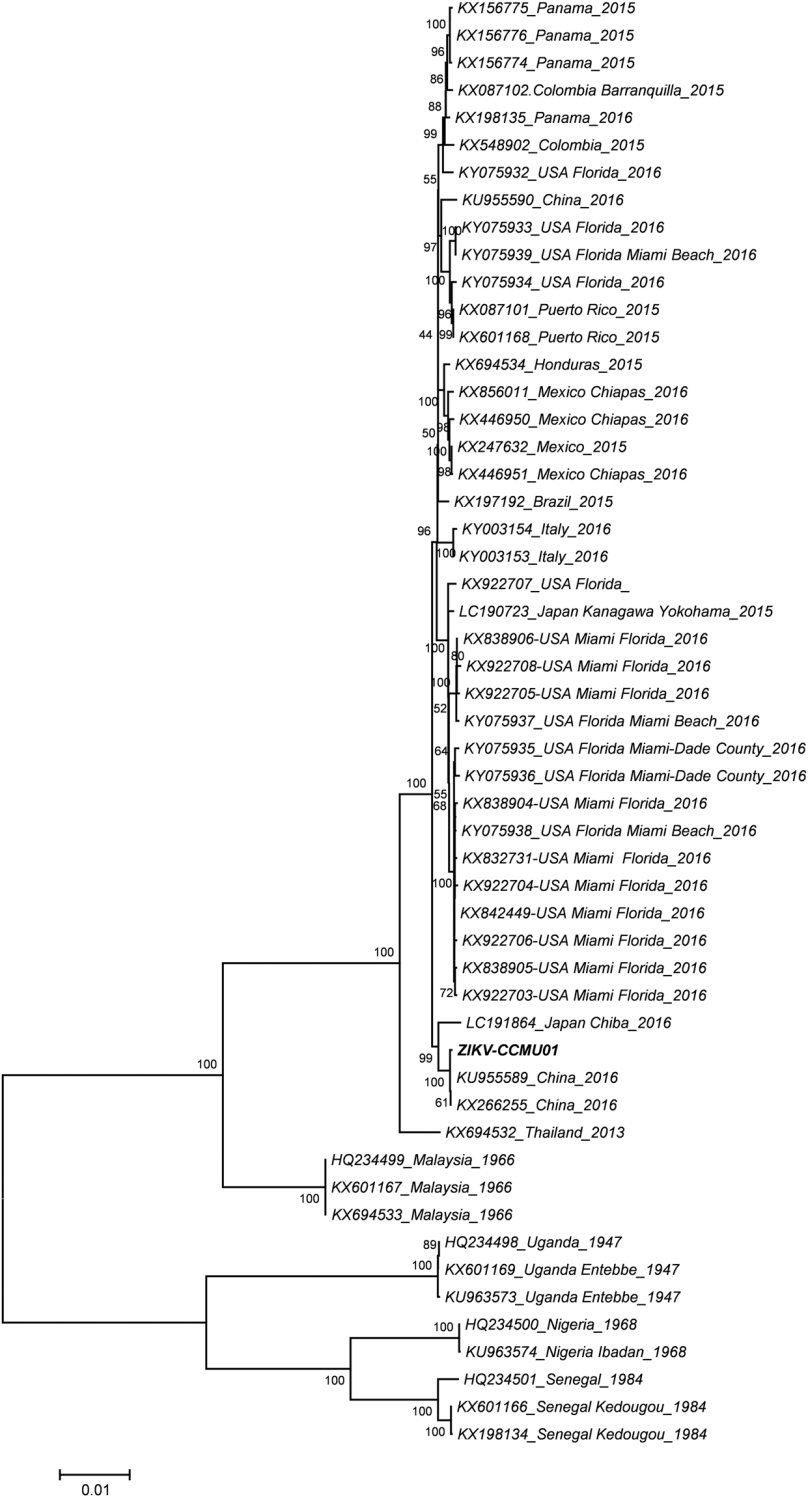
Phylogenetic analysis of Zika viruses. Phylogeny tree of the whole genome of ZIKV-CCMU01 and other 52 ZIKV strains registered in GenBank were constructed by Neighbor-joining method using MAGE 5 software.

To further characterize the ZIKV-CCMU01 isolate, phylogenetic tree was constructed and analyzed against 52 other ZIKV strains available (GenBank, 2017). Nucleotide sequence alignment showed that the ZIKV-CCMU01 isolate was clustered with the Asian lineage viruses, especially close to Z16006 (GenBank NO.: KU955589) and SZ_SMGC-1 (GenBank NO.: KX266255), that were isolated from Chinese returned from Fiji and Samoa, respectively (Fig. 3). ZIKV-CCMU01 shares 99.9% nucleotide sequence homology with these two other ZIKV strains.

### Pathogenicity of ZIKV-CCMU01 in suckling mice

**Figure 4:**
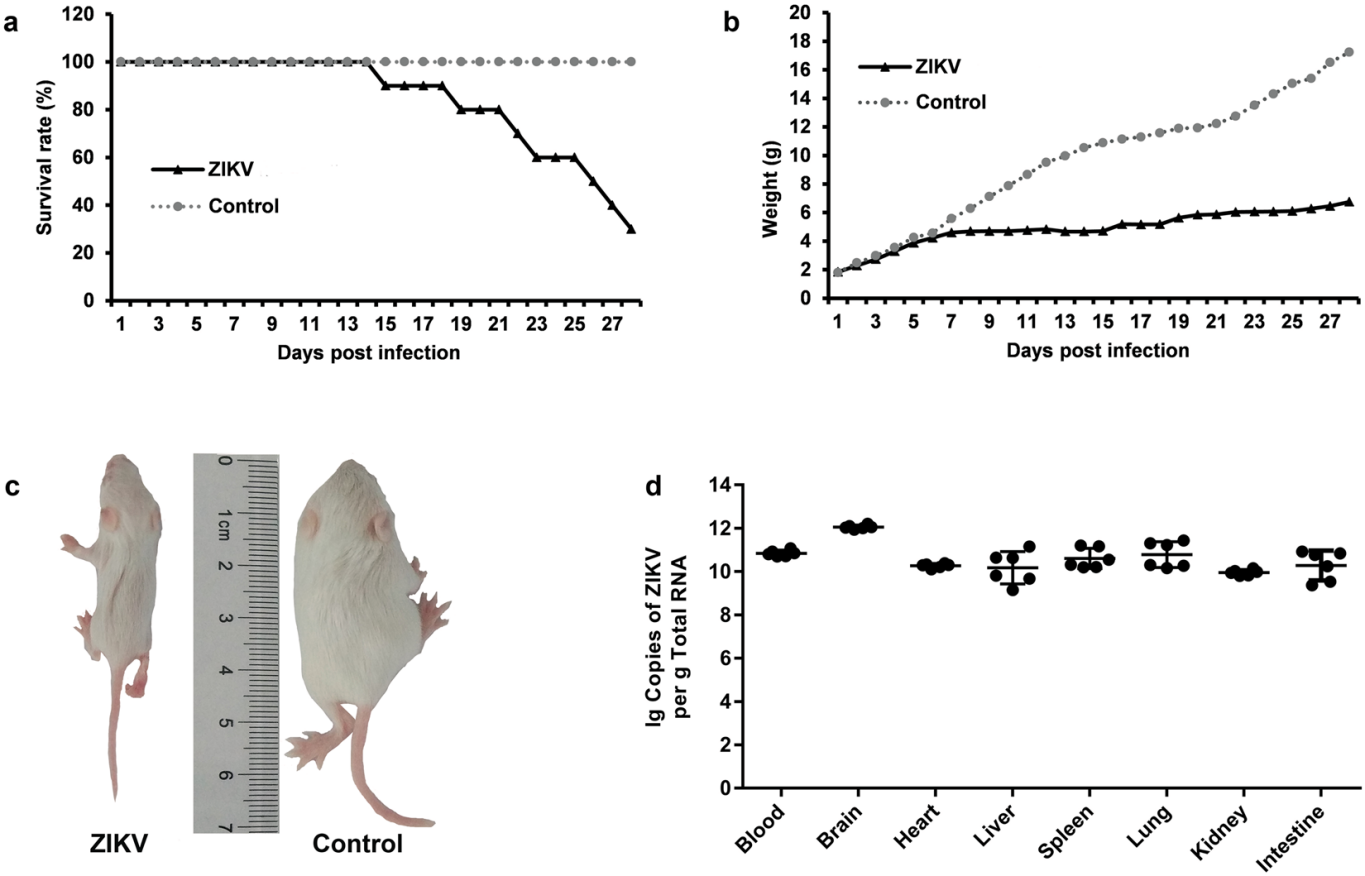
Pathogenic characteristics of ZIKV-CCMU01 in suckling mice. Pathogenicity of ZIKV-CCMU01 was assessed in Balb/c suckling mice by intracerebrally (i.c.) injection with 10 μL of viral solution (1,000 pfu) or 10 μL 0.9% NaCl solution (negative control). (**a**) Survival rates and (**b**) Changes of body weight were recorded from 1–28 dpi (n = 10). (**c**) Representative images of retarded mouse body-weight gain at 10 dpi. (**d**) Viral mRNA was detected in sera and major organs including brain, heart, liver, spleen, lung, intestine and kidney at 10 dpi by qRT-PCR (n = 10).

To investigative the pathogenesis of ZIKV-CCMU01, suckling mice born within 48 hours (hrs) were i.c. injected with 1,000 pfu/mouse of ZIKV-CCMU01 and then monitored for 28 days. From 8 dpi, mice showed weight loss, ruffled fur, lassitude and sluggish, and then become severely ill with signs of paralysis and moribundity. Death was observed from 10 dpi to 28 dpi. The total mortality was 70% (Fig. 4a). Accordingly, in infection mice, modest body weight gain was observed from 1 dpi to 7 dpi, but it almost stopped from 8 dpi onwards. At 28 dpi, infected mice had significantly lower average body weight than control mice (4.8 g vs. 16.5 g) (Fig. 4b,c). ZIKV was detected in serum, brain, heart, liver, spleen, lung, and kidney at 10 dpi, with titers ranged from 10^10^ to 10^12^ copies/g RNA (Fig. 4d). Taken together, these results indicated that ZIKV-CCMU01 is pathogenic to suckling mice.

## Discussion

Acute symptoms of ZIKV infection are observed in approximately 20% of cases between 3–12 days post infection^11^. Common clinical manifestations of ZIKV disease are fever, fatigue, headache, maculopapular rash, arthralgia, and myalgia^11^, which are similar to that of DF. However, in addition to neurological syndrome in adults and developmental complications in newborns, some unusual manifestations such as hearing loss and severe abdominal pain were also reported in ZIKV infection recently^11^. In this study, a patient with ZIKV disease developed pronounced liver dysfunction which had never been reported before and coagulation disorders that had only 16 reported cases since 2016.

The patient was healthy previously, with no medical history of liver disease and blood disease. But long-lasting and significantly elevated serum levels of ALT and AST as well as low ALB levels were observed after infection, indicating liver injury. Specifically, his AST and ALT levels were as high as 511.7 U/L and 329.6 U/L, which are 12.8 and 6.6 times higher than the upper limits of their respective normal range. To our knowledge, this is the first report of ZIKV patient with liver damage.

Moreover, markedly increased serum levels of LDH and α-HDBH during D6 to D13, and elevated CK levels during D6 to D9 were also observed in the patient. These enzymes are expressed extensively in body tissues such as blood cells, heart muscle and hepatocytes, and they are common markers of heart and liver injury^12, 13^. Therefore, in addition to the elevated levels of AST and ALT, the increased serum levels of LDH, α-HDBH and CK also indicated that the patient had extensive tissue and organ damage besides liver injury, suggesting ZIKV infection could damage several organs and cause their dysfunction. Consistently, a recent report indicated that patients with ZIKV infection had evidence of heart failure and development of dangerous heart arrhythmia^14^. Taken together, in combination with elevated serum levels of LDH, α-HDBH and CK, ZIKV may also cause heart problems. Therefore, clinicians should not only be aware of liver injury, but also pay attention to dysfunction of other organs such as heart in patients with ZIKV infection. Further study is needed to investigate the long-term effect of ZIKV infection on the function of major organs such as liver and cardiovascular system.

Coagulation disorder is another clinical characterization observed in this patient. It was manifested by low platelet counts, decreased Fbg and increased FDP levels. Fbg is synthesized in the liver by hepatocytes and it acts as the principal protein among human blood clotting factors. It is converted by thrombin into fibrin during blood clot formation. When plasmin breaks down fibrin, FDP is produced. Fbg level has been proposed as a predictor of hemorrhagic complications^15^. In this patient, lower levels of Fbg and higher levels of FDP may indicate a systemic activation of the clotting system and increased fibrinolysis trend which may be associated with liver injury. Thus, ZIKV infection may result in coagulopathy that may lead to bleeding.

Since the beginning of ZIKV epidemic in 2016 in the Americas, there were several reports describing cases with thrombocytopenic complications in ZIKV infection^16^. Among them, severe thrombocytopenia cases were found in whom the platelet counts were only 1 × 10^9^ or 2 × 10^9^ platelets/L. However, some of these patients have medical history of morbidly obese, untreated hypertension and hyperlipidemia, refractory acute immune mediated thrombocytopenia, or 7 months of pregnancy^17–20^. In contrast, our patient was previously healthy. ZIKV infection led to severe thrombocytopenia (dropped to 20 × 10^9^ platelets/L), and significantly decreased Fbg and increased FDP levels, indicating a severe blood coagulation disorders. Our results suggest that physicians should be aware of ZIKV associated blood coagulation dysfunction, and potential for bleeding.

ZIKV disease, DF and CHIKV fever (CHIKF) often share similar clinical symptoms such as fever, rash and conjunctivitis^4, 21^. Moreover, increased transaminases and thrombocytopenia are common clinical manifestations in DENV infection^22^. Therefore, differential diagnoses between ZIKV and DF or CHIKF are needed, especially in epidemic areas where DENV, CHIKV and ZIKV co-circulate.

In conclusion, in this study, we demonstrate a close link between liver injury and coagulation disorders and ZIKV infection by clinical laboratory blood tests, serology test, qRT-PCR and ZIKV identification from the patient’s serum. These results revealed rare pathogenic features in ZIKV infection and cautioned for vigilance by clinicians.

## Materials and Methods

### Case history

The patient was a previously healthy, 29 years old Chinese man who was returned from Cambodia after a business trip for 7 days. In Cambodia, he developed a fever (40.4 °C), chills and myalgia on 28th October, 2016, the day was defined as day 1 (D1) of fever onset. After being treated with antipyretic and antibiotic, his body temperature returned to normal temporarily. On D2 and D3, he developed fever (39 °C) again and felt very weak. Upon returning China, he was admitted to Beijing Ditan Hospital on November 2, 2016 (D6). Clinical signs and symptoms were recorded daily by a clinician, and serial blood samples were collected. To monitor functional change of major organs, routine blood tests and biochemistry tests including blood cell counts, liver function and coagulation tests, were performed in the hospital laboratories using Sysmex XE-5000™ Hematology Analyzer (TOA-DKK, Japan) and Hitachi H-7600-010 Biochemical Analyzer (Hitachi, Japan) according to standard protocols. The parameters that reflect tissue and organ injury, including serum ALT, AST, ALB, Fbg and FDP levels as well as CK, LDH and α-HDBH levels, were measured periodically. During the entire period of hospitalization, the patient mainly received glutathione and compound glycyrrhizin for reducing liver injury until being discharged from the hospital.

Serum sample obtained from the patient on D6 was tested for DENV^23^, CHIKV^24^ and ZIKV by qRT-PCR. Viral RNA was extracted from the serum sample using an *EasyPure*® Viral DNA/RNA Kit (TransGen, Beijing, China). qRT-PCR was performed with Quant one step qRT-PCR kit (Tiangen Biotech, Beijing, China) on an ABI 7500 Real-Time PCR System (Applied Biosystems, Foster City, CA, US) according to the manufacturer’s instruction. The primers of ZIKV are as follows: forward primer, 5′-TTGGGTTGTGTACGGAACCTG-3′ (nucleotides 678 to 698); reverse primer, 5′-GTGCTTTGTGTATTCTCTTGA-3′ (nucleotides 799 to 819).

Serology tests were also performed for detecting IgM and/or IgG against the following pathogens: ZIKV, DENV, CHIKV, HIV, HCMV, HSV-1, HSV-2, EBV, HBV, HCV and Treponema pallidum by enzyme-linked immunosorbent assay (ELISA), electrochemiluminescence immunoassay (ECLI) or chemiluminescent microparticle immunoassay (CMIA). The blood samples were also subjected to cultivation of bacteria and fungus to rule out other infections.

### Cells, virus and mice

The *Aedes albopictus* mosquito cell line (C6/36) was cultured at 28 °C in RPMI-1640 (Invitrogen, US) containing 10% fetal bovine serum (FBS, Gibco, Auckland, New Zealand). Vero cell was cultured at 37 °C in minimal essential medium (MEM, Invitrogen, US) supplemented with 5% FBS. DENV2 (Tr1751 strain) was isolated from a patient with DF, propagated in C6/36 cells and stored at −80 °C.

Pregnant Balb/c mice, purchased from the Academy of Military Medical Sciences (Beijing, China) were housed in a pathogen-free environment and suckling mice within 48 hrs after birth were used in experiments.

### Virus isolation and identification

Blood sample was obtained on D6 and centrifuged (Eppendorf, Hamburg, Germany) at 12,000 rpm at 4 °C for 30 min. Serum sample (0.2 ml) was inoculated in C6/36 cells, and then cultured at 28 °C for 6 days. The cell supernatant was collected and further cultured for 3 passages on C6/36 cells. At 6 day dpi of passage 3, virus infection was detected by IFA using 1:200 diluted anti-ZIKV polyclonal antibody produced in our laboratory as primary antibody, followed by 1:500 diluted FITC-conjugated anti-mouse IgG as secondary antibody. Images were taken by fluorescent inverted microscope (Olympus BX61, Japan). The viral titer in supernatants was determined by plaque assay on Vero cells under 1.2% methylcellulose overlay medium^25^.

### Plaque reduction neutralization test

Neutralizing antibodies (Nab) to ZIKV in serum samples obtained on D6 and D12 were assessed by plaque reduction neutralization test (PRNT) on Vero cells using a standard protocol^26^. In brief, after heat inactivation of complements, the serum samples were serially diluted and incubated with an equal volume of ZIKV (strain CAS-ZK01, Asian Lineage) containing approximately 200 pfu for 1 hrs. Then the mixtures were added to Vero cells for 1 hrs and followed by incubation with the overlay medium containing 1.2% methylcellulose at 37 °C for 5 days. Finally, crystal violet staining was used to visualize plaques formation. Human normal sera were used as negative control. The Nab titer was defined as the reciprocal of the maximum dilution of serum that yielded a PRNT_50_ compared with that of human normal serum controls.

### Sequence analysis

Total RNA was extracted from the infected cells using TRIzol reagents (Invitrogen, US). cDNA samples were prepared using TransScript-Uni One-Step gDNA Removal and cDNA Synthesis SuperMix Kit (TransGen Biotech, Beijing, China) according to the instructions. The virus genes were divided into 11 parts and amplified by PCR with specific primers (Supplementary Table 1). The viral genomes were sequenced by 3500xL Dx Genetic Analyzer CE-IVD (HITACHI, Japan) and analyzed against other 52 ZIKV strains registered in GenBank using MAGE 5 software. Phylogenetic tree of whole genomes was constructed using Neighbor-joining method.

### Pathogenicity of ZIKV in suckling mice

The pathogenicity of the isolate from the patient was assessed in suckling mice by intracerebrally (i.c.) injection of 10 μL viral solutions containing 1,000 pfu of ZIKV, or 10 μL 0.9% NaCl solution as negative control. Mice in each group were divided into two subgroups: in Subgroup I, blood samples and major organ samples including brain, heart, liver, spleen, lung, intestine and kidney were collected at 10 dpi for virus detection by qRT-PCR. A standard curve was established using ZIKV genome RNA (MR766) transcripted *in vitro*, kindly provided by Prof. Ai-Hua Zheng from Chinese Academy of Sciences (CAS) as standard template. The quantification of the copies of ZIKV mRNA in blood and major organ samples was determined as copy number per gram total RNA. The Subgroup II mice were monitored for 28 days during which body weight and mortality were recorded daily.

### Ethics statement

This study was performed in strict accordance with the institutional review board approval of the Ethical Committee of Beijing Ditan Hospital, China. Written informed consent was obtained from the patient. Human subject protection rules and regulations of China were strictly followed. All animal experiments were approved by and conducted in accordance with the guidelines set by the Institutional Animal Care and the Animal Ethics Committees of Capital Medical University.

## Electronic supplementary material

Supplementary Information
