# Supplementary material for: A unique case of human Zika virus infection in association with severe liver injury and coagulation disorders
Source: Sci Rep. 2017 Sep 12;7:11393. doi: 10.1038/s41598-017-11568-4 (PMC5595821; doi:10.1038/s41598-017-11568-4)
Supplement: Supplementary file 1 — Supplementary Information [file 41598_2017_11568_MOESM1_ESM.doc]

**A unique case of human Zika virus infection in association with severe liver injury and coagulation disorders**

Yanhua Wu^1#^, Xiaoyun Cui^1#^, Na Wu^2#^, Rui Song^3^, Wei Yang^1^, Wei Zhang^3^, Dongying Fan^1^, Zhihai Chen^3*^, Jing An^1,4*^

Supplementary table 1 Primers used for sequencing of ZIKV genome.

| Primer | Sequence (5'-3') |
| --- | --- |
| F1 | AGCAACAGTATCAACAGGTTTTATT |
| R1 | GACAACATCAACCCAAGTCCCACCT |
| F2 | GACTTTGTGGAAGGTATGTCAGGTG |
| R2 | AGTTCTCAGTGCTTTCAGTGATTAC |
| F3 | ATAACCGCTAACCCCGTAATCA |
| R3 | CTTCTCACTCTCAATCCAGTAGCCT |
| F4 | TGGATGGTGACACACTGAAGGA |
| R4 | GCAAAACCATTGATGAGAACCA |
| F5 | GCGTTGCTGGTATCTTTCATCTT |
| R5 | TGGGTGATGGCACTAACATAACTC |
| F6 | GGAGAGTGATAGGACTTTATGGCA |
| R6 | CTCCCTCAATGGCTGCTACTTT |
| F7 | ACAAAGTAGCAGCCATTGAGGG |
| R7 | TGAGTAGCAACCTATCATTAGCAGC |
| F8 | GACCACTTCATACAACAACTACTCC |
| R8 | GGTCTTTTTTCAAGCCAATCC |
| F9 | AAGTGAAAGGATACACAAAAGGAGG |
| R9 | CCTGAGTTCTCTCTCCCCATC |
| F10 | AGAGGAGAGTGCCAGAGTTGTGT |
| R10 | ATTTCGTAACTGGGGTCTTGTCT |
| F11 | GGAGAATGGATGACCACTGAAGA |
| R11 | AAACTCATGGAGTCTCTGGTCTTTC |
